# A Comparative Study Using Fluorescent Confocal Microscopy and Flow Cytometry to Evaluate Chondrocyte Viability in Human Osteochondral Allografts

**DOI:** 10.3390/bioengineering9100515

**Published:** 2022-09-29

**Authors:** Patricia López-Chicón, Tatiana Riba-Tietz, Oscar Fariñas, Pablo-Eduardo Gelber, Ricardo-Pedro Casaroli-Marano, Anna Vilarrodona

**Affiliations:** 1 Barcelona Tissue Bank (BTB)—Banc de Sang i Teixits (BST), 08005 Barcelona, Spain; 2 Biomedical Research Institute, 08005 Barcelona, Spain; 3 Department of Orthopaedic Surgery, Hospital de la Sta Creu i Sant Pau, Universitat Autònoma de Barcelona, 08005 Barcelona, Spain; 4 ICATME-Hospital Universitari Dexeus, Universitat Autònoma de Barcelona, 08005 Barcelona, Spain; 5 Department of Surgery, School of Medicine & Hospital Clinic de Barcelona, University of Barcelona, 08005 Barcelona, Spain; 6 Vall Hebron Institute of Research (VHIR), 08005 Barcelona, Spain

**Keywords:** osteoarthritis, osteochondral grafts, chondrocytes cell viability, graft preservation, confocal fluorescence microscopy, flow cytometry

## Abstract

The preservation conditions of fresh osteochondral allografts for clinical applications are critical due their objective: to transplant mature hyaline cartilage containing viable chondrocytes, maintaining their metabolic activity and also preserving the structural and functional characteristics of the extracellular matrix. The aim of the present study was to compare fluorescence confocal microscopy and flow cytometry techniques to evaluate the viability of the chondrocytes present in the osteochondral tissue, in order to determine their effectiveness and thus ensure reproducibility and robustness of the analysis. To this end, osteochondral allografts from human cadaveric donors were preserved at 4 °C for 3 weeks in a preservation medium supplemented with antibiotic and antifungal agents. Cell viability of chondrocytes was determined by monitoring the cartilage for 3 weeks of preservation by confocal fluorescence microscopy and flow cytometry, obtaining cell viabilities of 83.7 ± 2.6% and 55.8 ± 7.8% for week three, respectively. The confocal fluorescence microscopy approach is more advantageous and accurate, as it correlates better with actual cell viability values for monitoring osteochondral graft preservation, detecting only the cells that died a natural death associated with the preservation method.

## 1. Introduction

Cell viability is the main parameter to be evaluated when fresh osteochondral grafts are preserved for clinical applications [1,2,3]. Major studies have used fluorescent confocal microscopy to analyze the viability of chondrocytes in their natural three-dimensional arrangement, but only a few have compared different analytical techniques used to quantify this parameter [4,5]. Tetrazolium salt has been used to assess chondrocyte viability in cartilage preserved in several types of culture medium [6,7]. Other studies have assessed chondrocyte viability by fluorescent confocal microscopy, establishing it as a better technique than the use of colorimetric biochemical methods such as lactate dehydrogenase (LDH), calcein AM-ethidium homodimer-1 or trypan blue [5,8]. Depending on the technique used to evaluate cell viability, the percentages of living or dead cells will differ due to the enzymes responsible for the activation of calcein AM or LDH, because biologically, these molecules have different metabolic pathways. Moreover, the mathematical approximations used to analyze the experimental data obtained with the various techniques differ: a hemocytometer is used to quantify the number of viable cells stained with trypan blue, while a custom macro is used to quantify histological or confocal fluorescent images. In our study, we compare flow cytometry and confocal fluorescence microscopy techniques. Flow cytometry is considered to be an invasive technique, since the method used for sample preparation involves extracellular matrix enzymatic digestion to isolate the cells in order to quantify the percentage of live/dead cells. However, other studies have shown that imaging techniques overestimate the number of viable cells [5]. Furthermore, calcein AM and 7-AAD viability probes are incubated within the native cartilage by passive transport from the dye buffer solution to the chondrocytes embedded in the extracellular matrix, without modifying the native cartilage. Calcein AM produces fluorescence due to the esterase activity of viable cells, while 7-AAD is incorporated into nucleic acids of non-viable cells as a result of plasma membrane compromise.

There is currently no systematic test for assessing cell viability in osteochondral grafts, nor studies that compare the use of flow cytometry with traditional confocal fluorescent microscopy. In the present study, femoral condyles were preserved, cell viability was monitored, and the percentage of viable chondrocytes was quantified using semi-quantitative confocal fluorescence microscopy and quantitative flow cytometry techniques. Establishing a reference method to assess viability would allow standard assessment of the quality of osteochondral allografts, facilitating the safe distribution of tissues for transplantation in young patients with large osteochondral defects who currently do not have effective alternative treatment to recover functionality of the affected joints.

The aim of our study was to compare these two analytical techniques for quantitative determination of cell viability of cartilage chondrocytes present in osteochondral allografts for clinical use. We hypothesize that flow cytometry analysis will allow, using a simpler technology, a higher degree of system characterization for the detection of live, dead and early apoptotic cells compared with confocal fluorescence microscopy, which is unable to distinguish between the double staining that occurs between live and early apoptotic cells.

## 2. Materials and Methods

### 2.1. Ethical Considerations

Human samples were obtained, processed, and analyzed according to current guidelines in relation to the collection and preservation of human tissues for clinical use (EEC regulations 2004/23/CE and 2006/17/CE) and in accordance with the protocol and legal requirements for the use of biological samples and biomedical research in Spain (Law 14/2007 and RD 1716/2011). In addition, the acquisition, processing, and preservation of the tissues used in this study were carried out in accordance with Spanish law on the development and applications of organ transplants (RD 9/2014). All the information provided before donation, together with informed consent, guaranteed that the samples obtained were to be used for clinical applications and/or applied research. The use, protection, communication, and transfer of personal data complied with local regulations (Law 15/1999).

### 2.2. Reagents

The following reagents were used in the study: Ringer’s lactate (Fresenius Kabi, IL, USA, cat # 628339.4), calcein AM (Fisher Scientific SL, MA, USA, cat # 10462052), 7-AAD (BD Biosciences, NJ, USA, cat # 559925 ), Annexin V (Annexin V–APC, BD Biosciences, NJ, USA, cat # 550474), Vybrant^®®^ DyeCycleTM Ruby Stain (Invitrogen, MA, USA, cat # V10273), 0.9% NaCl (Braun, Kronberg, Germany, cat # 3570470), PBS (Gibco, MA, USA, cat # 14190-094), tobramycin (Medicon HealthCare, Maharashtra, India, cat # 672722.5), vancomycin (Laboratorios Reig Jofré, Barcelona, Spain, cat # 606390.3), amphotericin A (XalabarderFarma, Barcelona, Spain, cat # 820152268), cotrimoxazole (Almirall, Barcelona, Spain, cat # 656754.8), pronase (Merck Life Science, Darmstadt, Germany, cat # 10165921001), type II collagenase (Sigma-Aldrich, Darmstadt, Germany, cat # C1764-50MG), DMEM (Gibco, cat # 619965059), FBS (Biowest, Nuaillé, France, cat # S1860-500), HEPES (Sigma-Aldrich, cat # H0887-20ML), Glutamine (Biowest, cat # X0550-100), and a penicillin/streptomycin/amphotericin cocktail (Antibiotic Antimycotic Solution, Merck, Darmstadt, Germany, cat # A5955-20ML).

### 2.3. Osteochondral Tissue Processing

Osteochondral grafts were harvested from deceased donors within 24 h after death and stored at 4 °C. The study used 12 femoral condyles from three human cadaveric donors, obtained after consent to be used for scientific purposes. Femoral condyles were processed in class B cleanrooms under a class A laminar flow cabinet in our classified facilities (Barcelona Tissue Bank (BTB)—Banc de Sang i Teixits (BST); www.bancsang.net (accessed on 27 August 2022). Soft tissues adjacent to the joint area were removed and the bone area of the graft was washed with 0.9% NaCl to remove any remaining blood and fat residues. Condyles were stored for three weeks in physiological medium (Ringer’s lactate) supplemented with an antibiotic cocktail (tobramycin, vancomycin, cotrimoxazole) and anti-fungal agent (amphotericin A).

### 2.4. Cell Viability by Flow Cytometry

For the flow cytometry analysis, cartilage samples were obtained by sagittal cutting (400 mg) and chopped into small fragments. Pre-digestion was carried out with 1 mg/mL pronase dissolved in culture medium (DMEM, 1% HEPES, 1% Glutamine, 1% penicillin/streptomycin/amphotericin supplement). Cartilage fragments were incubated for 1h at 37 °C with gentle agitation. A second digestion was subsequently performed by adding 3 mg/mL type II collagenase dissolved in complete culture medium (DMEM, 10% FBS, 1% HEPES, 1% glutamine, 1% penicillin/streptomycin/amphotericin supplement), and incubated for 3 h at 37 °C with gentle agitation. After digestion, the cell suspension was filtered through a 70-µm pore membrane filter to remove undigested cartilage fragments. The cell suspension was then centrifuged at 2400 rpm for 5 min and the pellet was resuspended in PBS and divided equally into 6 aliquots. These were incubated at room temperature (RT) in darkness with the following probes: (i) calcein AM (4 µM for 30 min), (ii) 7-AAD (0.5 µg/mL for 10 min), (iii) Annexin V (0.5 µg/mL for 10 min), and (iv) Vybrant^®®^ DyeCycleTM Ruby Stain (5 µM for 15 min). The cell fluorescence signal (5 × 10^5^ cells/mL and 5000 events per measurement) was acquired using a BD LSR Fortessa cytometer (Becton Dickinson, San José, CA, USA) using the following lasers and detectors: (i) Blu-ray/violet, 405 nm and 50 mW; (ii) Pacific blue, 488 nm and 50 mW; (iii) FITC, 640 nm and 40 mW; and (iv) Alexa Fluor, 647 nm and 50 mW. The data obtained were analyzed using FACS Diva software (Becton Dickinson).

### 2.5. Cell Viability by Confocal Fluorescence Microscopy

For the confocal fluorescence microscopy assay, fragments of cartilage were obtained by sagittal cutting of the cartilage into 2 × 2 mm pieces. Samples were first incubated with 1 mL of 4 µM calcein AM dissolved in PBS for 30 min at RT in darkness with soft stirring. They were then washed twice with PBS and incubated with 1 mL of 0.5 µg/mL 7-AAD for 10 min at RT in darkness with soft stirring. They were then washed twice with PBS. The cell fluorescence signal was acquired by a Leica TCS SP5 confocal inverted microscope (Leica Microsystems, Mannheim, Germany) on a DMI6000 stand, with the Leica Application Suite Advanced Fluorescence software (Leica LAS AF, version 2.6.3.8173), using a 10×/0.4 NA HC PL APO objective. Calcein images were taken with the 488 nm laser line and emission detection between 495 and 550 nm, taking two averages. Images of the 7-AAD were taken with the 561 nm laser line and emission detection between 574 and 666 nm, taking two averages. The transmitted light channel was used to set the limits of the tissue block in the tile scan area, but not for image acquisition. The channels of 3D stacks were taken sequentially in stack-by-stack acquisition mode. Scanner speed was set at 600 Hz in bidirectional mode. The cartilage fragment was scanned from the surface to the deepest area adjacent to the bone, and cartilage images of the superficial, middle, and deep cartilage area were acquired. The images were bidirectional, captured with a 10× magnification objective, at 1024 × 1024-pixel resolution (speed of 600 Hz) using digital capture software. Images were analyzed using ImageJ Fiji software (1.52p version; https://imagej.net (accessed on 27 August 2022) through hand-made custom macros that quantified live and dead cells individually. Projection of an image of all z-stack planes together was split into 2 channels, to analyze the calcein and 7-AAD signals, respectively. A threshold value was selected in the histogram to define the value from which the signal would be counted as a particle. The segmentation tool was then applied to separate all sets of cells and differentiate them as a single unit. Finally, the particle analysis tool was applied to count the particles collected in the green and red channels, corresponding to the living and dead cells, respectively.

### 2.6. Statistical Analysis

Experiments were performed with cartilage from femoral condyles of at least three different donors, and each assay was performed with three technical replicates. The percentage of cell viability is presented as the mean ± standard error of the mean (MD ± SEM) for: number of living cells, dead cells, and apoptotic cells, referring in all cases to the total cell numbers. ANOVA analysis followed by Dunnett’s post hoc test was used for comparisons; *p* values less than 0.05 were considered statistically significant. The statistics were determined using commercial software (GraphPad Prism version 5.00 Windows, GraphPad Software, San Diego, CA, USA, www.graphpad.com (accessed on 27 August 2022).

## 3. Results

### 3.1. Cell Characterization by Flow Cytometry

**Figure 1:**
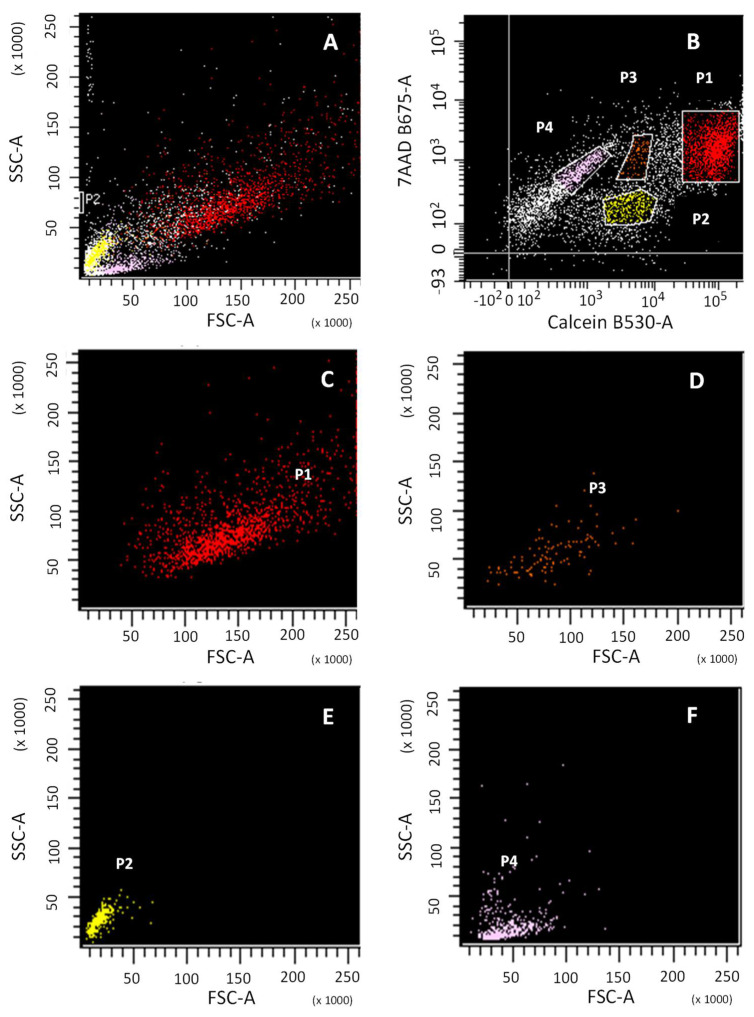
Flow cytometry analysis of cell viability of digested cartilage. Cell size (FSC) vs. cellular complexity (SSC) signals of the total population of: total cartilage digested (**A**); sub-population P1 of viable cells, calcein+ (**C**); sub-population P3 of viable cells, calcein+ (**D**); sub-population P2, calcein- (**E**); sub-population P4, calcein- (**F**). Calcein and 7-AAD signal of the total population (**B**).

Cartilage digested and analyzed by flow cytometry showed cells of different sizes, each incorporating a different amount of calcein AM, as well as small artefacts that also incorporated it. To ensure that the detected calcein signal corresponded to a cell or artefact, cell characterization was carried out by flow cytometry. Calcein AM and 7-AAD were used to assess the percentage of viable and non-viable chondrocytes, respectively, following enzymatic digestion of cartilage (Figure 1).

Four cell subpopulations were obtained (Figure 1B; P1, P2, P3, P4) with different patterns of emission for calcein and 7-AAD. Subpopulations P1, P2 and P3 were calcein positive, while P4 was not. The Forward SCatter/Side SCatter (FSC/SSC) pattern (Figure 1A) provides information about cell size and granularity, respectively. FSC/SSC was able to identify calcein-positive subpopulations P1 and P3, corresponding to viable cells (Figure 1C,D, respectively) and calcein-negative subpopulations P2 and P4, corresponding to cellular debris and non-viable cells (Figure 1E,F, respectively).

**Figure 2:**
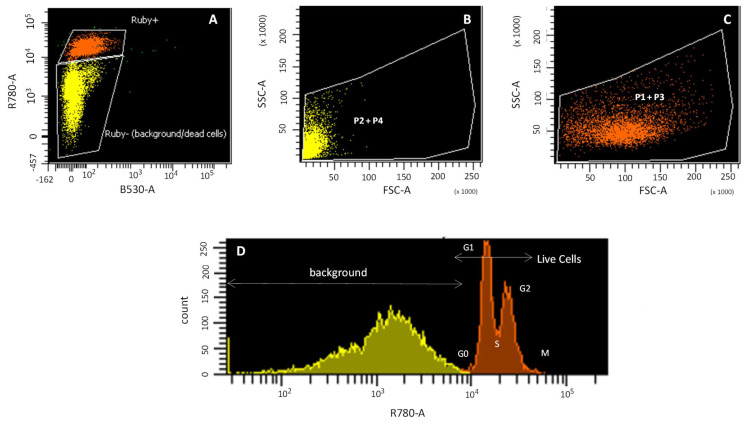
Cell cycle study by flow cytometry. Ruby (R780-A) signal (Ruby+ orange and Ruby-yellow) (**A**); cell size (FSC) vs. cellular complexity (SSC) of P2 + P4 populations (**B**); cell size (FSC) vs. cellular complexity (SSC) of P1 + P3 populations (**C**); Histogram of Ruby fluorescence signal (**D**).

The cell cycle study with the Vybrant^®®^ DyeCycleTM Ruby Stain (Ruby), without calcein, shows the DNA concentration, which allows the chondrocyte cell cycle pattern to be described, versus the FSC/SSC pattern, which is used to describe the cell morphology (Figure 2). The Ruby plot showed Ruby-positive (orange) and Ruby-negative (yellow) populations (Figure 2A). The Ruby-negative population corresponds to dead cells with highly degraded DNA and cellular debris (Figure 2B; yellow), with a pattern consistent with background in FSC/SSC and therefore with P2 and P4 populations (Figure 1E,F). The Ruby-positive population corresponds to those cells with intact DNA (Figure 2C; orange) with a size/cellular complexity pattern consistent with a living cell in FSC/SSC and therefore P1 and P3 populations (Figure 1C,D). Cellular debris of apoptotic and/or necrotic cells has highly fragmented DNA (Figure 1D) and an FSC/SSC pattern typical of non-viable cells (Figure 1E,F). The Ruby histogram showed two populations: (i) a double peak, corresponding to those cells that are within the cell cycle (Figure 2D; orange); and (ii) a broad band, corresponding to apoptotic or necrotic cells (Figure 2D; yellow).

P1 and P3 populations (Figure 2C) are thus considered living cells, because (i) both FSC/SSC plots show a cell size pattern, although P3 has a smaller cell size than P1; (ii) the positive staining for Ruby indicates that they are within the cell cycle; and (iii) both populations are calcein positive, although P3 incorporates less calcein than P1. However, the P2 and P4 populations (Figure 2B) are not considered living cells, since for both populations, the FSC/SSC plot shows small particles that are not within the cell cycle, as indicated by their negative Ruby signal.

### 3.2. Cell Viability Calculation and Apoptosis

**Figure 3:**
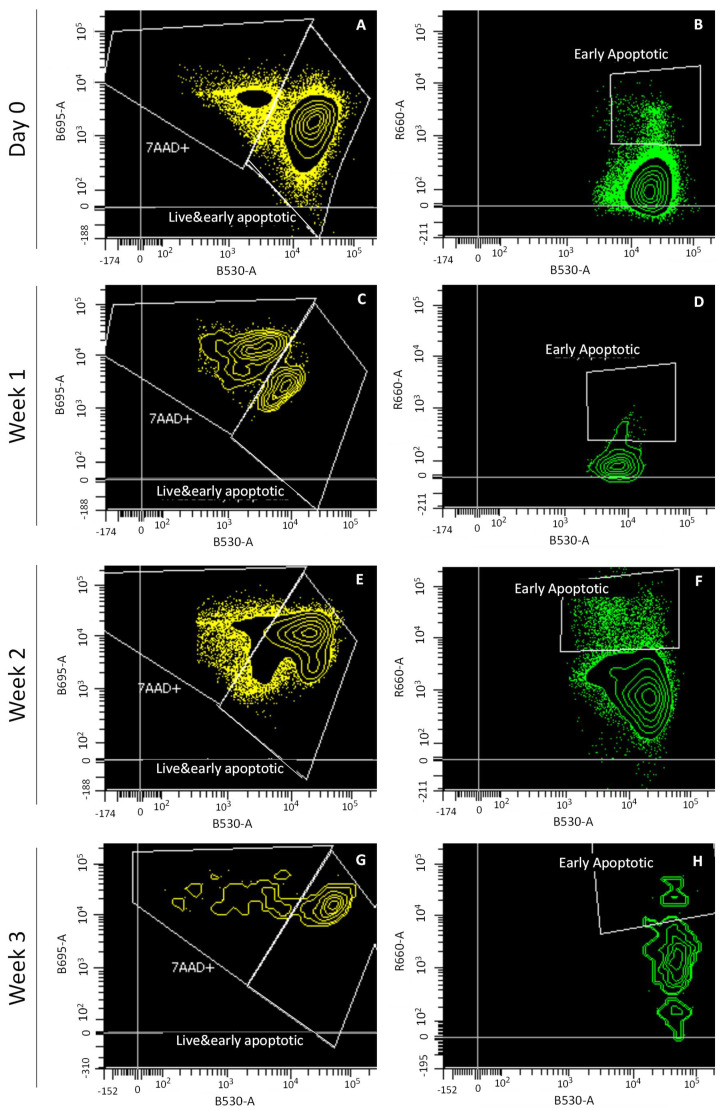
Flow cytometry analysis of cell viability of cartilage preserved at day 0, and at weeks 1, 2 and 3. Channels shown: calcein (B530-A), 7-AAD (B695-A) and Annexin V (R660-A). Plots shown: day 0 (**A**,**B**), week 1 (**C**,**D**), week 2 (**E**,**F**), and week 3 (**G**,**H**).

Flow cytometry analysis was performed to quantify, from P1 and P3 populations, the chondrocytes from cartilage preserved at day 0, and 1, 2 and 3 weeks. Flow cytometry plots show living, early apoptotic and dead cells after labeling with calcein AM, Annexin V and 7-AAD (Figure 3).

**Figure 4:**
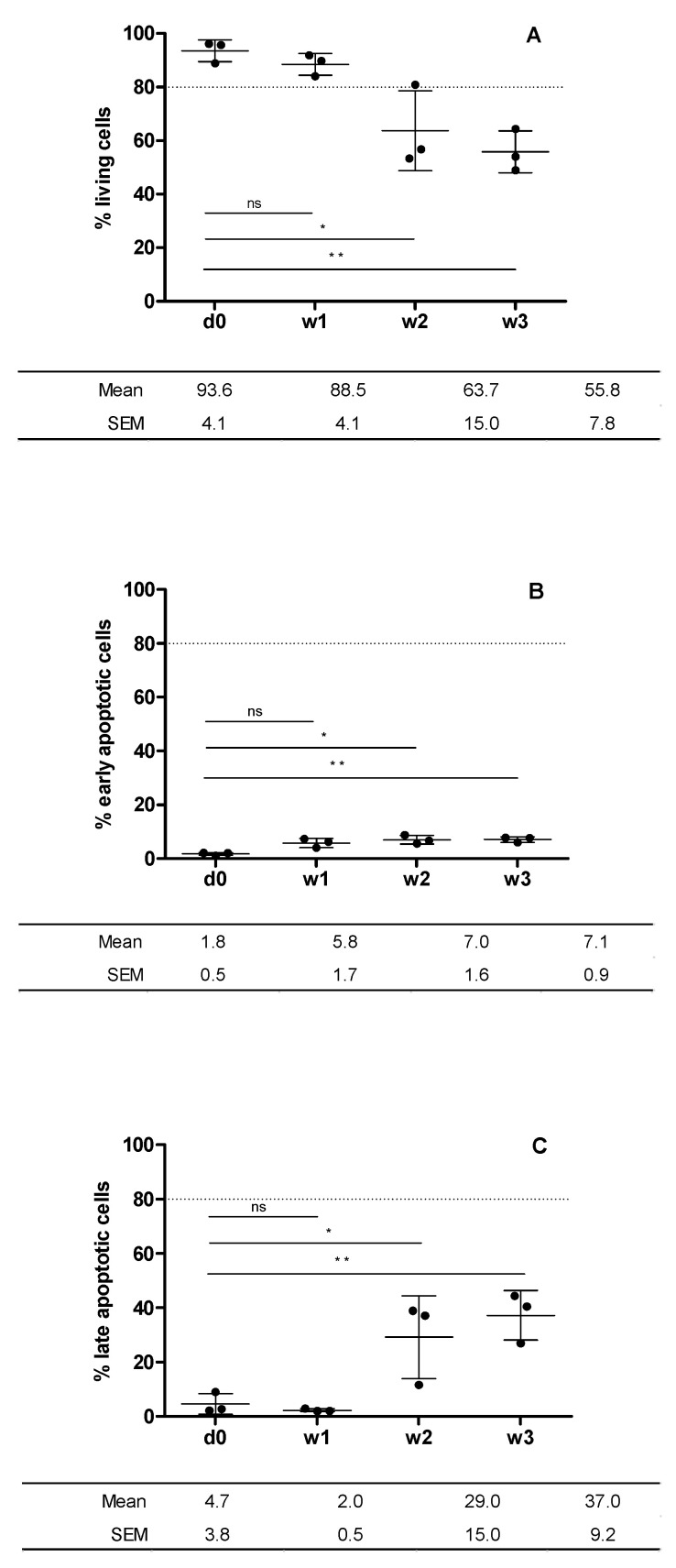
Cell viability of cartilage preserved at day 0 and at weeks 1, 2 and 3. Plotted from data obtained from flow cytometry analysis (Figure 3). Living cells (calcein+) (**A**); early apoptotic cells (Annexin V + 0 (**B**); late apoptotic cells (7-AAD+) (**C**). Statistical test: results are shown as mean ± SEM (n = 3) after performing non-parametric ANOVA followed by Dunnett’s post test. Differences between results are considered statically significant at a value of *p* < 0.05: * *p* < 0.05, ** *p* < 0.01. Differences between results considered non-statistically significant are indicated as ns.

As stated by the percentages viability over time presented in Figure 4, cell viability showed a very low ratio of early apoptotic cells with respect to the total number of cells (Figure 4B). The number of early apoptotic cells was negligible with respect to the total number of living cells (Figure 4A). Cell viability only remained above 80% during week 1, since at weeks 2 and 3, it decreased to around 55.8 ± 7.8%, which corresponds to the increase in the number of late apoptotic cells in the last two weeks of graft preservation (Figure 4C).

### 3.3. Cell Viability by Confocal Fluorescence Microscopy

**Figure 5:**
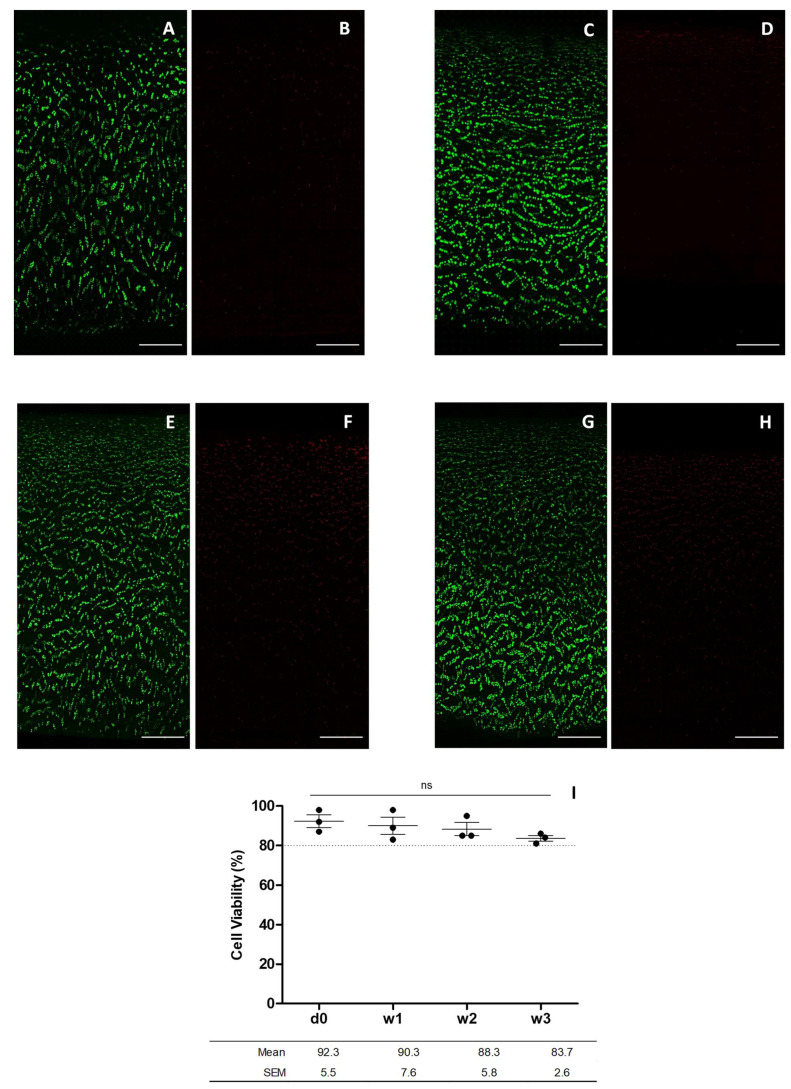
Images for chondrocyte viability obtained by confocal microscopy after incubation with calcein AM and 7-AAD. The images show the cell viability at day 0 (**A**,**B**), week 1 (**C**,**D**), week 2 (**E**,**F**), and week 3 (**G**,**H**) (scale bar corresponds to 100 µm) and cell viability of cartilage preserved at day 0, and 1, 2 and 3 weeks plotted from data obtained from confocal fluorescence microscopy (**I**). Statistical test: results are shown as mean ± SEM (n = 3) after performing non-parametric ANOVA followed by Dunnett’s post hoc test. Differences between results are considered non-statistically significant (ns).

Cell viability was determined by confocal fluorescence microscopy of chondrocytes embedded in a slice of human articular cartilage scanned from the surface to the deepest area adjacent to the bone. Fluorescent images of chondrocytes in the superficial, intermediate, and deep area of the cartilage were acquired. The same flow cytometry donors were assessed and their cell viability monitored at day 0, and at 1, 2 and 3 weeks of graft preservation. Fluorescent cell viability images showed the cartilage stained with calcein AM and 7-AAD, to detect live and dead cells, respectively (Figure 5A–H). Plotted data was obtained from the cell viability confocal fluorescence images thorough cell counting with ImageJ software. Cell viability was 92.3 ± 5.5%, 90.3 ± 7.6%, 88.3 ± 5.8% and 83.7 ± 2.6% for day 0, and weeks 1, 2 and 3, respectively (Figure 5I).

## 4. Discussion

Our study compared two methods for assessing cell viability in osteochondral grafts. As a result, the cell viability of chondrocytes measured by confocal fluorescence microscopy and flow cytometry was 83.7 ± 2.6% and 55.8 ± 7.8% for week three, respectively. Contrary to our hypothesis, confocal fluorescence microscopy approach is more advantageous and accurate, as it correlates better with actual cell viability values for monitoring osteochondral graft preservation, detecting only the cells that died a natural death associated with the preservation method.

Although several scientific studies have explored the best method for preserving fresh osteochondral grafts, no consensus has yet been reached regarding the ideal preservation media [4,9,10,11,12,13,14,15,16,17]. Considering these previous studies as a whole, several preservation media appear to be suitable for maintaining the viability of osteochondral allografts, but it is unclear which medium facilitates better chondrocyte survival, owing to the heterogeneity of results among studies. We hypothesize that these different cell viability results are due to the complexity of analyzing fluorescent confocal microscopy images, since there is no systematic standardized method to determine the cell count using microscopy images. Several approaches have been used to determine chondrocyte viability, such as LDH histochemical staining, calcein AM-ethidium homodimer-1 (CaAM-EthH) staining measured with confocal microscopy and cartilage digestion followed by trypan blue assay [5]. In relation to the quantification of living and dead cells, the study showed that CaAM-EthH was the only technique to overestimate the cell numbers; however, it was the most appropriate for providing valuable information related with the native cartilage. Furthermore, when LDH histochemical staining was compared with calcein AM-ethidium homodimer-1 staining, measured with confocal microscopy, it resulted in a greater percentage of viable cells analyzed with confocal microscopy than with LDH histochemical staining [8]. In flow cytometry, the cell solution resulting from digested cartilage is mixed with calcein AM and 7-AAD, which selectively enter living and dead cells, respectively, thus enabling their quantification. The final percentage of cell viability corresponds to the sum of the cells that died due to the stress suffered during the digestion process plus the number of cells that died due to the graft preservation process itself. In contrast, the confocal fluorescent microscopy technique overcomes these limitations methods used maintain its original histological architecture. The cell viability results obtained differed depending on the analysis technique used. Fluorescent confocal microscopy presented 83.7 ± 2.6% viability compared with 55.8 ± 7.8% viability obtained using flow cytometry, after three weeks’ tissue preservation. Flow cytometry results were lower, as previous enzymatic digestion of the cartilage matrix causes cell death inherent to the sample preparation, but not values determined compared with the real cell viability, due to natural causes associated with the preservation method used. In contrast, analysis using confocal fluorescence microscopy allows the preserved osteochondral allografts to maintain their cell viability as described previously [18,19,20]. Evaluation of cell viability by confocal fluorescence imaging enables analysis of cartilage samples in their real state of preservation because cartilage does not require significant processing steps prior to analysis. Conversely, the post-analysis methodology used to count living and dead cells is less systematic, producing variations between studies [4,9,10,11,12,13,14,15,16,17]. Mathematical treatment of fluorescence images to obtain a live-dead ratio involves a hand-made design of macro templates that include particle cluster fragmentation, and it establishes a fluorescence signal threshold from which the signal is considered a particle (and counted as a cell) or is discarded. Other scientific studies support this evidence, concluding that overestimation of the total cell count by confocal fluorescence microscopy can be explained due to random factors derived from inaccurate calculation of the effective volume of the scanned cartilage fragment, the refractive index of the cartilaginous tissue, and the water content and the angle of the scans by which the fluorescence images are acquired [5]. Thus, quantification of cell viability using the laser scanning confocal microscopy technique can give poorly reproducible results in cell detection as a result of the water content, extracellular matrix, and the optical properties of the cartilage sample.

Aside from the method used to perform post analysis, another point that has been taken into account is the ability of the technique to distinguish between living and apoptotic cells. Calcein AM is a probe that has the ability to penetrate the plasma membrane of cells. In contrast, 7-AAD is a large molecular size probe that is able to intercalate to the nucleic acids of non-viable cells with a compromised extracellular membrane. When intercalated, the 7-AAD/DNA complex allows the quantification of non-viable cells. Annexin V is a specific marker of apoptotic cells that binds to the phosphatidylserine protein when it translocates from the inside to the outside of the plasma membrane. This occurs when the cell enters in apoptosis, since phosphatidylserine has the function of signaling to the macrophages that the apoptotic cell must be phagocytized [21]. When intercalated, the Annexin V/phosphatidylserine complex permits the quantification of apoptotic cells. In addition, in the flow cytometry technique, chondrocytes can be labeled with a cell cycle probe as a control for the correct allocation of subpopulations of living or apoptotic cells. This analysis evaluates the phase of the cell cycle in which the cells are found by staining them with the Ruby probe because of its ability to penetrate the extracellular membrane of viable cells and intercalate with their DNA. When viable cells are in one of the different phases of the cell cycle (G0/G1, S or G2/M), a DNA fluorescence spectrum is obtained after irradiation. The signal results in a double peak emission pattern of different intensity depending on the amount of DNA contained in the cells, according to the phase of the cell cycle in which the cells are found. In contrast, apoptotic cells have their DNA so fragmented that Ruby staining is negative. Furthermore, necrotic cells appear in the initial broadband, corresponding to low DNA concentrations. Calcein AM produces double staining as this probe is incorporated by both living and early apoptotic cells, but the post-analysis method does not have the ability to differentiate between this double staining. Thus, it counts a fraction of the cell population that becomes apoptotic really early as a living cell, since the former also produce stain positive for calcein. In contrast, flow cytometry detects early apoptotic cells in the Ruby histogram area, delimiting the wide peak of degraded DNA and the double peak corresponding to the cell cycle. In addition, it is also possible to differentiate early apoptotic cells, as these stain positive for Annexin V. However, although flow cytometry makes it possible to distinguish between live and early apoptotic cells, the cell viability result is lower compared with the real value, since this technique counts dead and early apoptotic cells produced by the enzymatic digestion of the sample and not by the viability resulting from the graft preservation method.

Each analytical technique has its advantages and limitations, and it must be assumed that, depending on the analytical technique used and the parameters fixed by the analyst, the cell viability threshold value obtained after preserving the allograft will be different. Confocal fluorescence microscopy is more advantageous or convenient to flow cytometry in terms of simplicity and required laboratory time, since the sample does not require pre-treatment. This means that the cell viability results obtained correlate better with the state of viability of the cells at the time of preservation. In addition, it provides information on cell distribution and morphology. However, it has two limitations: (1) the inability to discern apoptotic cells, including them as living cells and therefore overestimating counting them as living; and (2) the subjectivity of the post-analysis method used is a function of the hand-made macros used, which produces a deviation between the results found in different studies. The flow cytometry technique is superior in terms of accuracy, since it can distinguish between living, dead and apoptotic cells. However, it has more limitations than confocal fluorescence microscopy. The results obtained using this technique do not accurately reflect the real cell viability values, as it underestimates the number of living cells by counting cells affected by enzymatic digestion of the sample, which are not associated with the natural death due to the cartilage preservation method, as dead or apoptotic. In addition, it provides no information about cell distribution or morphology. It is less advantageous in terms of time and simplicity, since it requires a long protocol for digestion of the cartilage fragments, as well as incubation of the different fluorescence probes. Both stages require a total time of approximately 6 h until cytometry measurements can be performed. In contrast, the confocal fluorescence microscopy technique only requires about 45 min of sample preparation until the images can be acquired.

## 5. Conclusions

Overall, although the post-analysis method used is complex, the confocal fluorescence microscopy approach is more advantageous and accurate than flow cytometry. It correlates better with real cell viability values because it does not require additional digestion steps that likely alter the osteochondral matrix tissue. Additionally, confocal fluorescence microscopy is methodologically simpler than flow cytometry in terms of sample preparation, despite the complexity of the post-analysis needed. Moreover, flow cytometry approach overestimates the number of dead cells as it is not possible to differentiate between cell death related to preservation method and the procedure itself. Regardless of the limitations of each technique, confocal fluorescence microscopy is an effective technique to evaluate cell viability and to assure sufficient quality of osteochondral grafts for clinical applications.

## Data Availability

No new data were created or analyzed in this study. Data sharing is not applicable to this article.
